# Analysis of post-traumatic growth status and its influencing factors in patients with facial palsy

**DOI:** 10.1186/s41016-018-0145-4

**Published:** 2018-12-18

**Authors:** Qian Li, Pengwei Lu, Yanzhu Fan, Lei Wang, Fei Yao, Diya Su

**Affiliations:** 1 Department of Neurosurgery, Beijing Tiantan Hospital, Capital Medical University, 6 Tiantan Xili, Beijing, 100050 China; 2 China National Clinical Research Center for Neurological Diseases (NCRC-ND), Beijing, 100050 China

**Keywords:** Facial nerve palsy, Post-traumatic growth, Coping style, Social support

## Abstract

**Background:**

Facial nerve paralysis in patients occurs in varying degrees of self-image disorders, both physically and mentally, resulting in low self-esteem, anxiety, depression, and even suicide; however, there were few researches on psychological problems in facial palsy patients at home and abroad. This study’s objective was to investigate post-traumatic growth (PTG) in facial nerve palsy patients and analyze its influencing factors.

**Methods:**

Using the convenience sampling method, a total of 47 patients with facial nerve paralysis were enrolled in the current study between June 1, 2016, and May 31, 2017. Post-traumatic growth rating scale was utilized to investigate the post-traumatic growth of these patients, and factors influencing patients’ post-traumatic growth were analyzed through collecting the general sociological information, disease-related information, simple coping style questionnaire, and social support rating scale.

**Results:**

The total score of post-traumatic growth in patients with facial nerve paralysis was mean (M) = 63.1, standard deviation (SD) = 19.14. The ranking of five dimensional scores from high to low was as follows: new possibilities, personal strength enhancement, appreciation of life, mental changes, and improvement of relationships with others. Multiple linear regression analysis showed that six variables, namely, the personality type, duration with facial nerve paralysis, and four coping styles, consisting of three types of positive coping styles and one negative coping style, could explain 71.6% of the total post-traumatic growth score.

**Conclusions:**

Post-traumatic growth in facial nerve palsy patients is moderate. The personality type of patients, the disease duration, and the coping style are the primary influencing factors. Therefore, clinical staffs should perform personalized nursing protocol and psychological intervention for facial nerve palsy patients to reduce their negative mood, improve their compliance with treatment, and help them recover more rapidly.

## Background

Trauma is defined as an event that threatens or injures a life or mind. In post-traumatic psychological research, much attention has been paid to domestic- and foreign-related trauma due to the high incidence of post-traumatic stress disorder (PTSD) in wounded people. In recent years, studies have shown that trauma does not result only in PTSD but also in easy-to-ignore positive changes. This concept of post-traumatic growth (PTG) was proposed by Tedeschi and Calhoun in as early as 1996, which refers to the individual’s struggle after an encounter with negative traumatic life events to adapt sufficiently to experience a positive change. At present, the research of PTG has expanded from natural disasters, wars, terrorist attacks, accidents, maltreatment, and other issues in the field of medicine. PTG’s research includes cancer, AIDS, myocardial infarction, chronic renal failure, and rheumatoid arthritis [1]. Physical appearance is important to individuals. Facial nerve paralysis (or facial paralysis disease) is a type of nervous system disease, characterized by unilateral or bilateral facial muscle movement functional disorder, which is often caused by tumors, trauma, infection, surgeries at the cerebello-pontine angle, and so on. The disease typically results from benign lesions but can variously affect changes in facial function and thus seriously affect the patient’s quality of life. Accordingly, facial nerve paralysis patients are suffering from trauma from varying degrees of self-image disorders, both physically and mentally, resulting in low self-esteem, anxiety, depression, and even suicide. Clinicians should not ignore facial nerve paralysis-related psychological problems [2, 3]. Therefore, the aim of this study is to analyze the relevant influencing factors of PTG in facial nerve palsy patients and investigate the clinical intervention measures to help them better cope with this disease and improve their quality of life.

## Methods

### Patient enrollment

Using the method of convenience sampling, patients with facial nerve palsy who were admitted from June 1, 2016, to May 31, 2017 to Beijing Tiantan hospital were selected. The inclusion criteria were as follows: aged 18–62 years, conscious, emotionally stable, no cognitive impairment, no communication barriers, and voluntary participation in this study. Patients who could not properly understand the scale were excluded.

## Demographic information

Using a self-designed questionnaire, information describing the patients’ gender, age, education level, religious belief, working condition, marital status, income, living area, personality type, past experiences, cause(s) of facial nerve paralysis, duration time, degree of disease, surgical treatment, and ability to wash and dress themselves was collected.

## PTG rating scale

Post-traumatic growth inventory (PTGI) by the American scholar Tedeschi and Calhoun [4] includes five dimensions with a total of 21 items: new possibilities (5 items), relationships with others (7 items), personal power (4 items), appreciation of life (3 items), and change of spirit (2 items). Using the Likert 6-grade evaluation method, from “post-trauma, no change” to “post-trauma, with a very large change,” each item was scored from 0 to 5 points, for a total score of 0 to 105 points, with a higher score in dictating a higher level of PTG. The total Cronbach’s alpha coefficient was 0.9, and the Cronbach’s alpha coefficient of each component ranged from 0.67–0.85.

### Simple coping style questionnaire (SCSQ)

Using the approach of Xie’s revision of the SCSQ [5] resulted in a questionnaire ascertaining the positive and negative coping styles of two subscales, containing a total of 20 items. We used a four-level scoring method, namely, “do not use,” 0 points; “occasionally use,” 1 point; “sometimes use,” 2 points; and “often use,” 3 points. The object of study was evaluated by self-assessment, with the coping subscales reflecting the individual’s response in cases of stress. The total Cronbach’s alpha of the scale was 0.89, the Cronbach’s alpha coefficient of the active component table was 0.89, and the Cronbach’s alpha coefficient of the negative coping component table was 0.78. The use of the Chinese psychological assessment scale can more accurately reflect the psychological status of the Chinese people.

### Realizing social support rating scale

This study used Xiao’s method [6] to establish an understanding of the patient social support scale. The scale is divided into three dimensions, namely, objective support, subjective support, and the degree of social support, for a total of 10 entries. The internal consistency coefficient was 0.81, and the retesting reliability was 0.92. This scale provides strong reliability and is widely used in China.

### Data collection and quality control

We explained to patients who met the inclusion and exclusion criteria the purpose of the survey to collect the patient’s agreement to cooperate in completing the questionnaire. The questionnaire is distributed in an anonymous way and by the researcher himself in another way. We used unified instructions to complete the questionnaire, completed by the researchers on the spot. In this study, 50 questionnaires were distributed and 50 questionnaires were collected. The recovery rate was 100%, among which there were 3 invalid questionnaires and 47 valid questionnaires, for a 94% completion rate.

### Data analyses and statistical methods

In strict accordance with the statistical methods, the PTG rating scale score, the simple coping style questionnaire scores, and understanding social support rating scale measurement data that conformed to normal distributions were described as the means (M), standard deviation (SD), and non-normal data were described by the median and inter-quartile range.

Gender, education level, religious belief, working conditions, marital status, income, place of residence, personality type, past experience, the cause of the facial nerve paralysis, time, degree of disease classification with facial nerve paralysis, whether the patient had surgery, surgery time, and current degree of self-care were described as counting data using frequency percentiles.

The age of the patient was described using the mean number of the normal distribution and the mean value of the normal distribution. The non-normal data was described in terms of the median and quartile spacing.

The PTG level of patients with facial nerve paralysis was compared via an independent sample *t* test and signed-rank test. The relationship between the PTG of facial nerve palsy and other numerical variables was analyzed. With the PTG-scale total score and each dimension score as the dependent variable, the single-factor analysis results, literature review, clinical experience, facial nerve paralysis patient demographic data, disease, coping styles, and social support factors were analyzed to identify the related factors of PTG using multiple linear regression analysis.

All the statistical analyses were performed using SPSS 20.0 software.

## Results

### Demographic and disease-related information

A total of 47 patients with facial nerve palsy were enrolled in this study, and the ages ranged from 22 to 62 years old (M = 40.2, SD = 10.41). Clinical characteristics of these patients were shown in Table 1.

**Table 1:** Demographic and medical characteristics (*n* = 47)

Variables	*n* (%)
Gender	
Female	26 (55.3)
Male	21 (44.7)
Education level	
Low education (illiteracy/primary school)	7 (14.9)
Secondary education (secondary school/high school)	14 (29.8)
Higher education (university and above)	26 (55.3)
Religious belief	
Yes (Christian/Buddhism)	6 (12.8)
No	41 (87.2)
Marital status	
Married	37 (78.7)
Divorced, widowed, single	10 (21.3)
Monthly income	
Low income (RMB: 1000-3000)	25 (53.2)
Medium income (RMB:3001-5000)	11 (23.4)
High income (RMB: > 5001)	11 (23.4)
Residence	
City	29 (61.7)
Countryside	18 (38.3)
Personality type	
Extrovert	12 (25.5)
Middle	23 (48.9)
Introvert	12 (25.5)
Event	
Yes (accident/chronic disease/family misfortune)	6 (12.8)
No	41 (87.2)
Cause of facial paralysis	
Tumor in CPA area	26 (55.3)
Non-tumor (facial neuritis/trauma/other)	21 (44.7)
Time since diagnosis	
≤ 1 year	12 (25.5)
> 1 year	35 (74.5)
Degree	
Mild and moderate (level II/III/IV)	24 (51.1)
Severe (level V/VI)	23 (48.9)
Surgery	
Yes	37 (78.7)
Postoperative time < 1 year	15 (31.9)
Postoperative time 1–2 years	13 (27.7)
Postoperative time > 2 years	9 (19.1)
No	10 (21.3)
Self-care level	
Yes	46 (97.9)
No	1 (2.1)
Whether returned to work after rehabilitation	
Yes	32 (68.1)
No	15 (31.9)

### PTG in facial nerve palsy patients

**Fig. 1:**
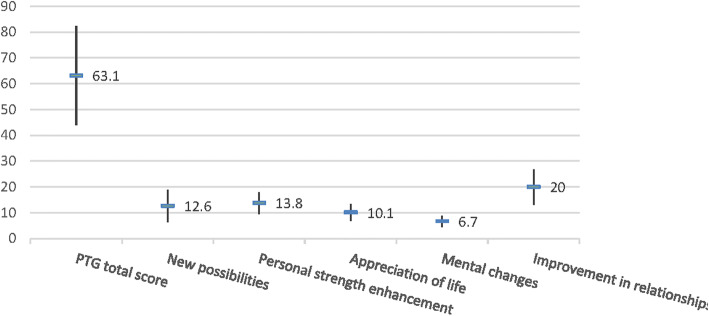
Post-traumatic growth total score for patients with facial nerve paralysis and five dimension score (*n* = 47, M, SD, score)

The average PTG score of 47 patients with facial nerve palsy was M = 63.1, SD = 19.14. The score of the five dimensions was as follows: relationships with others: M = 20.0, SD = 6.79; new possibilities: M = 12.6, SD = 6.16; personal strength: M = 13.8, SD = 4.20; mental change: M = 6.7, SD = 2.13; and appreciation of life: M = 10.1, SD = 3.26. The five dimensions ranked in order of average score from high to low were as follows: new possibilities, increased personal strength, appreciation of life, mental changes, and improvement of relationships with others (Fig. 1).

### Univariate analysis of PTG in patients with facial nerve palsy

#### Comparison of PTG of nerve palsy patients under different conditions

The results showed that the PTG associated with facial nerve palsy was not associated with age, sex, religious beliefs, marital status, other events, or facial paralysis. Facial nerve palsy in patients with PTG was associated with personality types, and the different personality types in order from high to low were as follows: extroverted, middle type, and introverted. The three different types of character for PTG had scores that were significantly different (*P* < 0.05). Patients with facial nerve palsy for more than 1 year were more likely to have higher growth scores than those who had the disease for 1 year, and the total difference in PTG was statistically significant (*P* < 0.05). Patients who had been treated with surgery had higher levels of PTG than those who did not have surgery, and the total difference in PTG was statistically significant (*P* < 0.05). The results showed that the total scores of postoperative growth in patients after surgery were higher than those of other patients after surgery, and the difference in scores was statistically significant (*P* < 0.05). In addition, the cause of facial paralysis was more commonly a cerebello-pontine angle (CPA) area tumor than trauma; although the *P* value comparing the difference in causes of facial paralysis was not less than 0.05, it was less than 0.1. Therefore, with an enlarged sample size, the result is likely to achieve statistical significance, see Table 2 for details.

**Table 2:** Comparison of post-traumatic growth of nerve palsy patients under different conditions (*n* = 47)

Variables	PTGI total score (M, SD)	*P*
Gender		0.701
Female	M = 62.2, SD = 20.03	
Male	M = 64.4, SD = 18.39	
Education level		0.619
Low education	M = 69.7, SD = 10.52	
Secondary education	M = 61.3, SD = 24.48	
Higher education	M = 62.4, SD = 17.93	
Religious belief		0.892
Yes	M = 62.2, SD = 20.98	
No	M = 63.3, SD = 19.13	
Marital status		0.529
Married	M = 62.2, SD = 19.56	
Divorced, widowed, single	M = 66.6, SD = 18.06	
Monthly income		0.764
Low income	M = 64.6, SD = 20.96	
Medium income	M = 63.6, SD = 13.44	
High income	M = 59.5, SD = 20.73	
Residence		0.633
City	M = 62.1, SD = 18.15	
Countryside	M = 64.9, SD = 21.06	
Personality type		0.024*
Extrovert	M = 74.3, SD = 16.57	
Middle	M = 62.5, SD = 17.83	
Introvert	M = 53.4, SD = 19.57	
Event		0.343
Yes	M = 56.2, SD = 22.20	
No	M = 64.2, SD = 18.74	
Cause of facial paralysis		0.087
Tumor in CPA area	M = 67.5, SD = 19.28	
Non-tumor	M = 57.9, SD = 18.01	
Time since diagnosis		0.034*
≤ 1 year	M = 53.2, SD = 21.90	
> 1 year	M = 66.6, SD = 17.12	
Degree		0.158
Mild and moderate (level II/III/IV)	M = 59.3, SD = 18.00	
Severe (level V/VI)	M = 67.2, SD = 19.84	
Surgery		0.016*
Yes	M = 66.6, SD = 18.02	
Postoperative time < 1 year	M = 63.3, SD = 23.57	0.030*
Postoperative time 1–2 years	M = 73.9, SD = 9.76	
Postoperative time > 2 years	M = 61.7, SD = 14.60	
No	M = 50.4, SD = 18.53	
Whether returning to work after habilitation		0.778
Yes	M = 63.7, SD = 16.68	
No	M = 62.0, SD = 24.20	
Self-care level		0.802
Yes	M = 63.1, SD = 19.34	
No	M = 68.0	

*: *p* < 0.05

#### Correlation analysis of coping style and social support and PTG in facial nerve paralysis patients

**Fig. 2:**
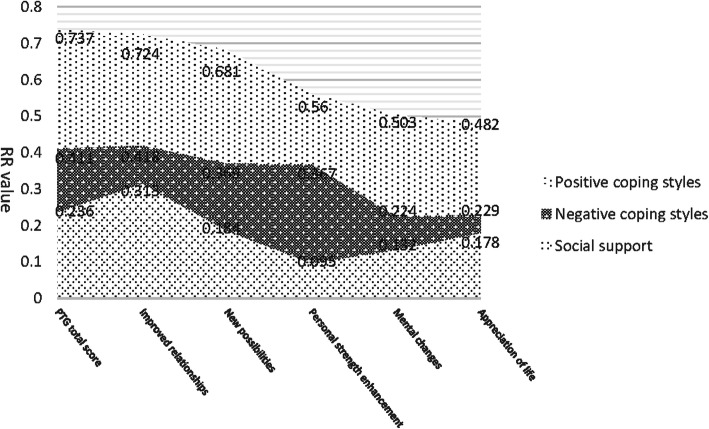
Correlation analysis of coping style and social support in facial nerve paralysis patients (*n* = 47, r)

The results are shown in Fig. 2.

#### Multi-variate analysis of PTG in patients with facial nerve palsy

In facial nerve paralysis patients with PTG, total score was considered the dependent variable and the results of single-factor analysis, literature review, clinical experience, demographic data, disease-related information, coping styles, and social support were considered independent variables in a multiple linear regression model, using the method of step-wise regression for variable selection. The independent variable assignments are shown in Table 3, and the results are shown in Table 4. Six variables were used in the regression equation, including the patient personality type, duration of facial nerve paralysis, and four coping styles, including three types of positive coping styles: “change the original practices or some of their problems,” “pursue hobbies and actively participate in recreational activities,” and “learn how to respond to similar challenging situations” and a negative response: “there may be some miracle that will change the status quo.” These six variables together accounted for 71.6% of the total PTG.

**Table 3:** Multiple linear regression analysis

Independent variable	Data input method
Education level	Low education = 1, secondary education = 2, higher education = 3
Age	According to the survey data entered
Marital status	Married = 1, divorced, widowed, single = 2
Personality type	Extrovert = 1, middle = 2, introvert = 3
Event	Yes = 1, no = 2
Causes of facial paralysis	Tumor in CPA area = 1, non-tumor = 2
Time since diagnosis	≤ 1 year = 1, > 1 year = 2
Degree	Mild and moderate = 1, severe = 2
Surgery	Yes = 1, no = 2
Postoperative time	< 1 year = 1, 1–2 years = 2, > 2 years = 3, no surgery = 4
Whether returning to work after rehabilitation	Yes = 1, no = 2
Coping styles for each dimension score	According to the survey data entered
Total score	According to the survey data entered

**Table 4:** Regression analysis of influencing factors of post-traumatic growth in patients with facial nerve palsy (*n* = 47)

Variables	*B*	*β*	*t*	*P*
Personality type	− 4.331	− 0.190	− 2.401	0.027
Time since diagnosis	8.880	0.204	2.570	0.014
Coping style (change the original practices or some of their problems)				
	7.884	0.390	3.671	0.001
Coping style (pursue hobbies and actively participate in recreational activities)				
	2.915	0.182	1.954	0.058
Coping style (learn how to respond to similar challenging situations)				
	6.940	0.292	2.718	0.010
Coping style (there may be some miracle that will change the status quo)				
	3.064	0.194	2.288	0.027

Note: The model *R*^2^ = 0.753, adjusted *R*^2^ = 0.716, *F* = 10.195, and *P* < 0.01

## Discussion

### PTG status in facial nerve palsy patients

This study of 47 patients with facial nerve paralysis had an average PTG score of M = 63.1, SD = 19.14 points, which was significantly higher than that previously reported by the study of parents of children with cancer (M = 44.9, SD = 16.9) points [7]. It was also significantly higher than that reported in a PTG study of patients with accidental traumatic fracture (M = 52.38, SD = 20.58) and in Operation Enduring Freedom (OEF) and Operation Iraqi Freedom (OIF) veterans following a major combat-related amputation [8] (M = 59.1, SD = 23). This study group included a patient who had suffered from accidental injury to the body and was in the recovery period. One reason for these differences may be that lesions in patients with facial nerve paralysis are only partial, namely, facial functional obstacles, and that the vast majority of the patients’ bodies have no damage and the body functions are not affected, meaning that self-care ability is not affected and that patients can live without having to rely on others. Another reason may be that facial nerve paralysis is a benign lesion and that facial nerve damage after the initial incident will not worsen. Facial muscles are amenable to exercise after facial atrophy following intervention, and appearance changes are likely through methods such as acupuncture, medication, exercise, and surgery [9]. In addition, 55.3% of patients with facial paralysis in this study had disease stemming from a tumor in the CPA area; 80% of CPA area tumors are acoustic neuromas, and the patients often have varying degrees of decline in hearing function and may also develop symptoms such as tinnitus and deafness. Although left facial nerve paralysis is a possible sequel following tumor excision surgery, the damage caused in this type of patients with acoustic neuroma has been lifted; thus, the overall level of PTG is higher than that of cancer, traumatic fracture, or accidental trauma in patients with damage to the body. For each dimensional item analyzed in this study, patients with facial nerve paralysis had PTG levels in the five dimensions from high to low as follows: new possibilities, enhancement of personal power, appreciation of life, mental changes, and improvement in relationships with others.

### Influencing factors for PTG in facial nerve paralysis patients

#### Patients with extroverted personalities had a high level of post-traumatic growth

Personality is an important component of psychological research and is also an important variable in the PTG model [10]. The results showed that different personality types were associated with statistically significant differences in PTG level, and the level of PTG from high to low regarding personalities was as follows: extroverted, middle type and introverted, and the PTG five dimension scores were consistent with these results. Analyzing the causes, extroverted people under stress are often more willing to express their emotions after the events and extroverted people, relative to relatively introverted people, are more optimistic. Previous studies have been reported on the relationship between personality characteristics and PTG. Research by Llewellyn et al. [11] found a positive correlation between a positive personality and PTG. Bostock, Sheikh, and Barton’s systematic review of the relationship between optimism and PTG in multiple personality traits also indicates a correlation between the two factors [12]. In addition, relatively extroverted people are more likely to have a friend to talk to, confide in, or ask for help when facing stress, which is a type of positive coping style. Scrignaro et al. [13] showed in 40 cases of cancer patients that taking positive and reasonable approaches can enhance PTG.

#### Patients with longer course of the disease have higher levels of PTG

Patients who have had facial nerve palsy for more than 1 year have higher levels of PTG than those who have had it for less than 1 year. Most of the patients in the face of disease undergo a starting period of denial, shock, and doubt after accepting the transition to the disease. In patients with a short course of disease, their mood is in a state of instability, and they often face a variety of other stresses, such as seeking medical treatment, undergoing treatment, and inspection. Studies have found that whether a traumatic event is perceived as too severe (more than the individual psychological status) leads to a positive psychological resource overdraft, impairing PTG in adolescents with chronic diseases [14]. The results from Senol-Durak & Ayvasik indicate that PTG in patients is significantly correlated with emotional response [15]. Over time, patients accept the fact that their face has changed, and the PTG level increases with the end of the process of seeking and undergoing medical treatment. In addition, the degree of facial paralysis can be improved after treatment with acupuncture, medication, and surgery, which may be the reason for the high level of PTG following the end of treatment seeking and acquisition.

#### Patients who have been treated with surgery have high levels of PTG

The surgical treatment of patients with facial nerve paralysis through a neurosurgery operation for facial nerve–hypoglossal nerve anastomosis allows restoration of the damaged facial nerve. Facial exercise training enables facial nerve function improvement or recovery [16]. The purpose of the operation is to bring new hope to patients a change in their psychological state. When surgery was performed in patients with PTG, the “new possibilities” dimension score difference was statistically significant (*P* = 0.021). There was also a statistically significant difference in “mental change” (*P* = 0.001). The study also found that PTG scores in patients 1 to 2 years after surgery (M = 73.9, SD = 9.76) were higher than those of postoperative patients within 1 year of surgery (M = 63.3, SD = 23.57). In patients further than 2 years postop (M = 61.7, SD = 14.60), the score difference was statistically significant (*P* = 0.030). Perhaps because of facial nerve paralysis after surgical treatment, the short-term effect is not significant, and through facial exercise after approximately 1 year, there appears to be a certain degree of recovery, which brings positive emotions. Several patients, due to various factors, do not see a significant improvement in their faces more than 2 years after the operation, and these patients frequently lose the positive psychological effects of recovery.

#### Patients with a low monthly income have high growth levels

The study showed that the total score of PTG in patients decreased with the increase in monthly income. Generally, educational level and monthly income were positively associated, and this study showed that patients with lower incomes had PTG scores that were higher than those of patients with medium or higher degrees of income. As for the potential reason, patients with higher incomes tend to have higher living standards; their demands are higher, and their image after facial paralysis occurs is associated with a more severe psychological gap than patients with lower requirements for image.

#### Patients with CPA tumors have high growth levels

In patients with facial nerve paralysis originating from tumors of the CPA area, the majority of tumors are acoustic neuromas, the disease duration is long, and patients often have different degrees of hearing damage and even prolonged symptoms such as headache, tinnitus, and deafness. Patients with intracranial tumor “time bomb” need to undergo surgery to alleviate prior uncomfortable symptoms, although most will experience postoperative facial nerve paralysis as a result. But the damage caused by this kind of acoustic neuroma in patients has been lessened, that is to say, even though patients went through with the surgery and suffer facial paralysis, there is improvement in the patient’s psychological well-being in the long course of the disease (a “bad thing” with a “good thing”). Beforehand, patients must be informed and understand the prognosis of the disease and notified by the doctor on what to expect before, during, and after the operation, including the various risks such as facial paralysis which may occur after surgery.. However, the facial paralysis in patients with no tumors is caused by accidental trauma and facial nerve inflammation. Suddenly, the patient has more stress and psychological trauma. It is more difficult to accept the result.

#### Positive coping style can help patients grow after trauma

Correlation analysis in this study shows that the PTG level and positive coping styles are positively correlated, and increasing research results suggest that positive coping styles and PTG are positively correlated [17]. Multi-variate linear regression analysis in this study included three types of positive coping styles in the regression equation (“change the original practices or some of their problems,” “seek a hobby to actively take part in sports,” and “draw lessons from others for similar difficult situations”); these styles were helpful for improving the level of the PTG. At the onset of the facial paralysis incident, clinical staff, through scientific health education, can target psychological interventions for the patients, such as encouraging patients to develop their own hobbies and to actively participate in sports activities after returning to a stable condition, helping patients to understand the coping styles that have helped other patients and to adjust, and encouraging patients to listen and talk to each other.

#### Negative coping style was positively correlated with the PTG level of patients with facial nerve paralysis

One study has shown that negative coping styles are unrelated to PTG [17]. Another study [18] showed in a longitudinal study of 50 bereaved adolescents who experienced trauma that negative coping styles of evasion after 6 years of PTG are correlated with new possibilities and personal power to change, which is consistent with the results of this study. The analysis found that the PTG total score was positively correlated with negative coping. The three dimensions of “improvement of relations with others,” “new possibilities,” and “personal power to change” were positively correlated with negative coping, and coping styles of “fantasy can be a miracle to change the status quo” were entered into the regression equation in the multiple linear regression analysis. In fact, the “fantasy miracle” belief itself can prolong the stressful event and bring pain to the patients, as we observe in the clinical long-term survival of patients who every day look forward to a miracle occurring. In clinical nursing, we can draw lessons from this type of approach; it is appropriate to provide information of previous treatment in patients with successful cases, providing hope for improvement in current patients. In addition, the study of coping styles identified “trying to take a break or rest, temporarily putting problems (trouble) aside,” and “comforting yourself” as belonging to the category of negative coping styles; however, these styles are not uniformly negative in real life, and putting questions aside and comforting themselves can, to some extent, increase the acceptance of stressful events and can therefore help patients achieve PTG.

#### The relationship between social support and facial nerve paralysis is not clear

Tedeschi and Calhoun [10] explicitly highlighted the correlation between social support and PTG, but that study showed that social support had no correlation with total scores of PTG. Only one dimension, namely, the improvement of relationships with others, was positively correlated. The origin of this relationship may be that patients with facial nerve paralysis experience changes in the external appearance and may develop self-image disorders that severely affect social functioning, during which patients often do not wish to meet with friends and family. The social support scale used in the investigation included several items related to the patients “colleagues,” and this study identified 10 patients (31.9%) with facial paralysis who did not continue to work after developing paralysis. The relationship between social support and the PTG in facial nerve palsy is recommended for further study.

## Conclusion

In summary, facial nerve palsy can exhibit positive psychological changes and achieve PTG in the face of physical and mental changes resulting from disease. The most significant aspect is the occurrence of new possibilities, which gives the clinical treatment staff nurse a goal of early psychological intervention in patients, with economic class playing a guiding role. Being extroverted, having facial paralysis caused by a CPA area tumor, and a longer disease duration are associated with relatively high PTG levels. This correlation suggests that clinical nurses, in giving patients nursing plans, should implement nursing measures aimed at patients who are introverted, have facial paralysis caused by sudden accidental trauma, have shorter disease durations, and have higher social status or even are public figures who pay more attention to their external image to provide early psychological intervention and emotional guidance to help patients adopt suitable ways to promote the level of PTG. Nurses should adopt personalized nursing plans, effectively reducing the patients’ negative mood to ensure better compliance with treatment and help patients recover both their physical and mental health more rapidly.

## Abbreviations

CPA: Cerebello-pontine angle
M: Means
PTG: Post-traumatic growth
PTGI: Post-traumatic growth inventory
PTSD: Post-traumatic stress disorder
SCSQ: Simple coping style questionnaire
SD: Standard deviation
